# Performance evaluation of ESR dosimeters under proton beam FLASH irradiation conditions

**DOI:** 10.1093/jrr/rraf054

**Published:** 2025-09-17

**Authors:** Jun Kumagai, Hiromitsu Iwata, Kenji Komaguchi, Chihiro Omachi, Toshiyuki Toshito, Masumi Umezawa, Masashi Yamada, Takashi Kondo

**Affiliations:** Institute of Materials and Systems for Sustainability, Nagoya University, Furo, Chikusa, Nagoya, Aichi, 464-8603, Japan; Department of Radiation Oncology, Nagoya Proton Therapy Center, Nagoya City University West Medical Center, 1-1-1 Hirate, Kita, Nagoya, Aichi, 462-8508, Japan; Graduate School of Advanced Science and Engineering, Hiroshima University, 1-4-1 Kagamiyama, Higashi-Hiroshima, Hiroshima 739-8527, Japan; Department of Proton Therapy Physics, Nagoya Proton Therapy Center, Nagoya City University West Medical Center, 1-1-1 Hirate, Kita, Nagoya, Aichi, 462-8508, Japan; Department of Proton Therapy Physics, Nagoya Proton Therapy Center, Nagoya City University West Medical Center, 1-1-1 Hirate, Kita, Nagoya, Aichi, 462-8508, Japan; Therapy System Business, Healthcare Business Group, Hitachi High-Tech Corporation, Kashiwa, Chiba, 277-0804, Japan; Therapy System Business, Healthcare Business Group, Hitachi High-Tech Corporation, Kashiwa, Chiba, 277-0804, Japan; Center for Low-Temperature Plasma Sciences, Nagoya University, Furo, Chikusa, Nagoya, Aichi, 464-8603, Japan

**Keywords:** ESR chemical dosimetry, lithium formate monohydride, alanine, FLASH, ultra-high dose rate, proton beam

## Abstract

Two chemical dosimeters, lithium formate monohydride (LFM) and L-alanine (ALA), were first evaluated under ultra-high dose rate (UHDR) proton irradiation conditions, known as ‘FLASH’, which has the potential to reduce the impact on normal tissue while effectively killing tumors, using electron spin resonance (ESR) spectroscopy. Both ALA and LFM demonstrated a significant linear increase in ESR peaks that correlated with the physical dose when comparing conventional radiation (CONV) to UHDR radiation. The relative effectiveness (RE: the ratio of the amount of free radicals produced by each type of proton irradiation to the amount produced by ^60^Co γ-ray irradiation) was determined for CONV, UHDR-Plateau and UHDR-Peak, yielding RE values of 0.849, 0.731 and 0.661 for LFM and 0.834, 0.692 and 0.624 for ALA, respectively. The decrease in RE values was likely due to the combination of UHDR and the increase of linear energy transfer (LET) to facilitate the recombination of radicals formed within the crystal during CONV and UHDR of proton beams. When using the height of the ESR central peak as an indicator of sensitivity, LFM was assessed to be ~20% more sensitive than ALA.

## INTRODUCTION

The potential for cancer treatment using ultra-high dose-rate irradiation (FLASH) to serve as a paradigm shift in radiotherapy is significant, as it has the capacity to mitigate the impact on normal tissue while demonstrating equivalent efficacy in targeting tumors as conventional radiotherapy [1, 2]. In the 1960s and 1970s, research was conducted on mammalian cells that had been irradiated with ultra-high dose rates (UHDRs) [3–6]. It has been confirmed that the increase in survival rate in normal tissue is better protected in comparison to normal dose rates. However, since these dose rates were not feasible for clinical radiation therapy, research into UHDR declined for a time. However, as accelerators have evolved, it has become physically possible to employ UHDR irradiation in radiation therapy. Favaudon *et al.* have termed the characteristic of reducing damage from UHDR irradiation the ‘FLASH effect’ [7], which has once again captured the attention of radiation therapy.

X-rays and γ-rays are commonly used in cancer treatment via external irradiation at normal dose rates; however, preparing photon beams under UHDR conditions is challenging, and, to our knowledge, there are no documented cases of their application in treatment. In contrast, electron beams can achieve high dose rates relatively easily, making UHDR irradiation feasible. For this reason, FLASH effects have mainly been observed in electron beam irradiation [1]. Electron beams generally have the highest dose on the surface of the irradiated object, and the dose weakens as it penetrates the object, so radiation therapy is mainly used for skin diseases. Vozenin *et al.* showed that when 34 Gy was irradiated to pig skin at a UHDR (300 Gy/s) and when 25 Gy was irradiated at a normal dose rate (0.08 Gy/s), skin fibrosis occurred after 36 weeks in the normal irradiation, but skin damage was reduced in the UHDR irradiation [8]. In 2019, a 75-year-old patient with skin-malignant T-cell lymphoma who had undergone several dozen radiation treatments was given clinical electron beam UHDR irradiation (167 Gy/s) and at 3 weeks, i.e. at the peak of the reactions, a grade 1 epithelitis (CTCAE (Common Terminology Criteria for Adverse Events) v5.0 [9]), along with a transient grade 1 edema (CTCAE v5.0) in soft tissues surrounding the tumor, was observed [10]. For body tumor irradiation, proton therapy is preferable to electron beam irradiation, as protons can achieve high dose-rate coverage at the Bragg peak in deeper tissues, while electrons can only target surface areas. If it is possible to achieve UHDR that satisfies the FLASH conditions in the plateau region of the proton beam passing through normal tissue and the Bragg peak at the tumor site, it may be possible to perform ideal radiation therapy that reduces the impact on normal tissue and kills tumors deep within the body. In order to confirm whether the desired dose has been irradiated when UHDR is performed using such FLASH effects, a dosimeter that can evaluate the absorbed dose is required.

Various types of dosimeters, such as ionization chambers, scintillators and electron spin resonance (ESR) chemical dosimeters, have been used in the fields of radiation chemistry, radiation biology and radiation medicine. In the early 1960s, it was discovered that when X-rays or electron beams are irradiated onto alanine (ALA) polycrystals, free radicals are produced in proportion to the dose, and they can be used as a chemical dosimeter [11–13]. In 1965, Ebert and others had already observed the number of free radicals produced by proton irradiation of ALA at 5–15.8 MeV [14], and, the following year, Henriksen *et al.* reported on free radicals generated by heavy ion irradiation of ALA [15, 16]. Many studies have been reported since 1985 on the evaluation of the performance of ALA as a chemical dosimeter in proton irradiation [17–24]. When ALA, represented as the chemical structure H_3_N^+^-CH(CH_3_)-COO^−^, is irradiated by ionizing radiation, the stable •CH(CH_3_)-COOH radical, forms through deamination from a protonated alanine radical anion. In addition to this radical, the H_3_N^+^-C(CH_3_)-COO^−^ radical also remains a second major radical component in irradiated ALA [25, 26]. The radicals, including the third major one formed in irradiated ALA, have been the subject of extensive debate, yet the subject remains unresolved [12, 25–33]. Recently, a report on the performance evaluation of ALA using electron beam UHDR irradiation was published [34]. However, to the best of our knowledge, no reports exist on the performance evaluation of ALA using proton beam UHDR irradiation.

Lithium formate monohydride (LFM), which produces stable CO_2_^•–^ radicals in the crystalline through ionizing radiation, is a relatively new ESR dosimeter compared to alanine. Since Lund *et al.* proposed that LFM could be a better ESR dosimeter than ALA [35], dosimetry studies of LFM applied for radiation therapy have been studied for X- or γ-ray [36, 37], proton-beam therapy [38, 39] and mixed radiation field [40–43], although fundamental studies on the radicals produced in LFM have also been examined [44–47]. There has been extensive research on LFM as a dosimeter; however, as far as we know, there are currently no reports of LFM under UHDR irradiation.

In this study, we irradiated LFM and ALA with proton beams under UHDR in both plateau (360–500 Gy/s) and peak regions (320–1418 Gy/s), reporting on the linearity to physical dose and relative effectiveness (RE) compared to γ-ray irradiation for these chemical dosimeters. Additionally, we irradiated them in the peak region at the dose rate of conventional (CONV) proton beam therapy (0.03 Gy/s). We present the results of an evaluation of LFM and ALA’s performance as dosimeters under FLASH conditions of proton beam.

## MATERIALS AND METHODS

### Materials

Lithium formate monohydride (LFM, TCI, >98.0%) and L-alanine (ALA; >99.0%, Kanto Chemical Co., Inc., Japan) were purchased and used for irradiation without further purification. As the standard for radical concentration, 2,2,6,6-tetramethylpiperidine-1-oxyl (TEMPO; > 98.0%, Sigma-Aldrich, USA) was used.

### Establishment of UHDR proton beam using synchrotron accelerator

Proton therapy device and clinical results have already been reported in detail [48–50]. The UHDR proton beam was generated using a synchrotron accelerator (PROBEAT III, Hitachi, Ltd, Tokyo, Japan). The detailed methodology for UHDR proton beam generation, including beam extraction control and dose rate management, has been described in our previous study [51]. In summary, UHDR conditions were achieved by extracting all protons within a single spill (10–50 ms) using high-frequency power at elevated levels. The extracted charge was monitored using a bunch monitor and verified with a Faraday cup, ensuring accurate dose control. The dose rate was defined as the maximum dose in the target volume divided by the spill width. To account for the limitations of conventional charge-based dose monitoring at high count rates, a time-based control method was employed for dose and dose rate adjustments. Although the repulsive force increases between protons at ultra-high dose rate irradiation in principle, each beam extraction condition is individually optimized in UHDR and CONV, and the effects are negligible in this experiment.

### Irradiation field and setup

A single-energy, non-scanning beam was used. Acrylonitrile–butadiene–styrene resin ridge filters were fabricated with a high-resolution 3D printer to form 1 cm-wide spread-out Bragg peaks (SOBPs) for UHDR. The ridge filter was installed 720 mm upstream from the isocenter, and the beam was transported to the scanning treatment room without modifying the transport conditions. For CONV, a 10 cm-wide SOBP was formed, and irradiation was performed at the center of the depth. Eighteen samples of capped 1 ml Eppendorf tubes, filled with ALA or LFM to a height of <10 mm, were arranged in a tube rack with three rows and six columns and placed at the center of the depth for irradiation. By removing one row (three tubes) for each irradiation, samples were prepared at cumulative doses of 5, 10, 20, 30, 40 and 50 Gy (peak region, SOBP, 0.03 Gy/s). For the UHDR study, a 96-well plate (Corning, New York, USA) was employed for dose measurement experiments. Each well of A1, A5 and A9 positions contained ~100 mg of ALA or LFM, ensuring a uniform depth of 5 mm from the bottom of the well. The plate was subjected to UHDR irradiation at both plateau and peak regions, as well as CONV irradiation at the peak region (SOBP). The specific doses delivered were as follows: UHDR irradiation (plateau region, 360–500 Gy/s): 4.5, 9.0, 13.5 and 18.0 Gy. UHDR irradiation (peak region, SOBP, 320–1418 Gy/s: 4.5, 9.0, 18.0, 27.1, 36.1 and 45.1 Gy. The dosimetry methodology, including the use of float glass, Gafchromic EBT-3 film and the Advanced Markus chamber, has been described in detail in our previous study [51]. It is guaranteed that the dose is properly delivered within an error range of 3–5%.

### Profile of linear energy transfer

**Fig. 1:**
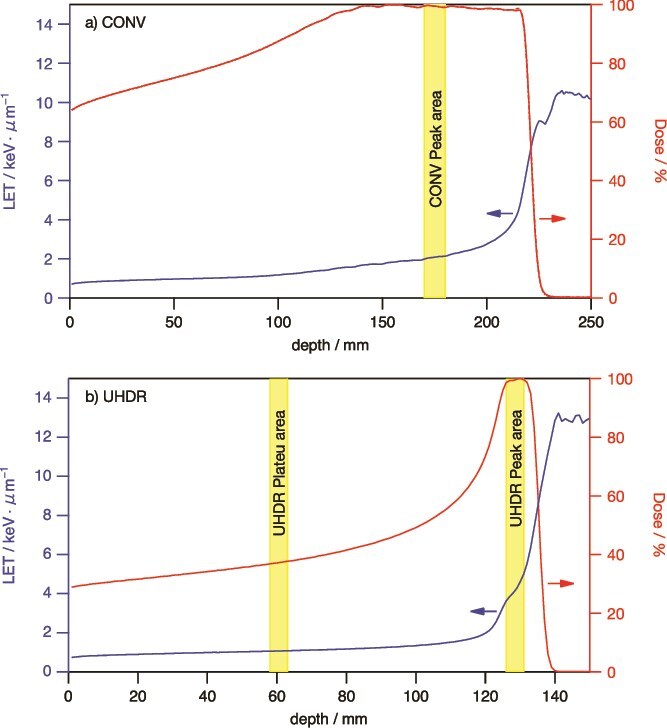
The calculated physical dose (red lines) and LET (blue lines) as a function of the depth for incident protons at the SORB for CONV (a) and UHDR (b) sample positions.

In our previous study [52], we reported that the LETs in the plateau region and the center of the SOBP (10 cm width) for CONV irradiation were identical. However, in this study, since the SOBP width in UHDR is narrower (1 cm), we conducted additional LET simulations to evaluate potential differences. LET calculations were performed using Monte Carlo simulations, as described in our previous study [52]. In summary, the LET was calculated using the Geant4 (10.4-patch 2 version) Monte Carlo simulation, with a binary cascade model for hadron and ion inelastic processes and a range cut of 1 km to suppress delta-ray production. The simulation setup, including the beam geometry and energy settings, was equivalent to those used in previous biological experiments. The dose-averaged LET was determined based on the track length and deposited energy within 1-mm grid spacing. LET values from primary and subsequently produced protons (including secondary and tertiary) were calculated, while secondary electrons and other ion particles were disregarded. The depth profile of LET and dose are plotted in Fig. 1 for both CONV and UHDR irradiation.

### Irradiation of γ-rays

LFM or ALA powder crystals placed in 1.0 ml Eppendorf tubes were irradiated with γ-rays at the Co-60 irradiation facility of Nagoya University, Japan. The doses for LFM were 4.6, 9.3, 18.5, 27.8, 37.1 and 46.4 Gy, while for ALA, they were 5.0, 10.0, 20.0, 30.0, 40.0 and 50.0, respectively. The dose rate for the γ-irradiation was ~0.16 Gy/s.

### ESR measurements

The irradiated samples were stored in a desiccator of <4.0% humidity, and they were introduced into the ESR tube to a height of 1 cm, weighed (typically ca. 80 mg), capped and measured at room temperature. ESR spectra were measured by a JEOL JES-RE1X spectrometer, Japan at Nagoya University. Microwave frequency was monitored by a Hewlett-Packard HP 53150A CW microwave counter. ESR measurement was completed within 3 days of irradiation for all samples. The ESR parameters for the measurements are summarized in Table 1.

**Table 1:** ESR parameters for the measurements of LFM and ALA crystals

Parameters	LFM	ALA
Microwave power (mW)	1.0	1.0
Magnetic field (mT)	337 ± 7.5	337 ± 10
Field modulation width (mT)	0.5	0.5
Field modulation frequency (kHz)	100	100
Amplitude of signal	200	500
Time constant (s)	0.1	0.3
Sweep time (min)	4	8
Accumulation times	2	2

The samples were measured both in the absence and presence of the standard Mn digital marker (JEOL ES-DM1). The standard sample was inserted into the cavity to the same depth (500 for ES-DM1), ensuring that it did not affect the peak intensity or peak position of the sample. The average decrease in the measured microwave frequency when the Mn marker was inserted was 114 kHz, which corresponds to a low-field shift of 0.004 mT in the peak position, but this corresponds to only a 0.05% shift in the ±7.5 mT sweep width, and there was no clear difference in the peak position or peak intensity of the actual observed spectrum. The difference between the maximum and minimum heights of the first derivative of the fourth Mn peak, calculated from the difference between the two spectra, served as an indicator of sensitivity. An ESR spectrum without the Mn marker was double-integrated to obtain the peak area of the sample. A 110 μl (a volume that results in a height of 1.0 cm in the ESR tube) of TEMPO solution (5.24 μM) dissolved in tetrachloroethylene introduced into the ESR tube and sealed was used for the radical concentration standard.

## RESULTS AND DISCUSSION

The LET and dose depth profiles for the CONV and UHDR irradiation of the newly recalculated proton beam are presented in Fig. 1a and b, respectively. For CONV irradiation, the sample is located at the center of the area where the Bragg peak is sufficiently flat in SOBP and the thickness of the sample is within the 10 mm frame indicated in yellow in the figure. The LET of CONV irradiation received by the sample is estimated to be ~2.0 keV μm^−1^. UHDR irradiation at the plateau and peak regions of the sample irradiation were around 60 and 128 mm depths, indicated by the yellow frame with LETs of ~1.0 and 4.0 keV μm^−1^. There is a rather wide distribution of LET (3.6 ~ 5.0 keV μm^−1^) depending on the depth for UHDR-Peak irradiation.

**Fig. 2:**
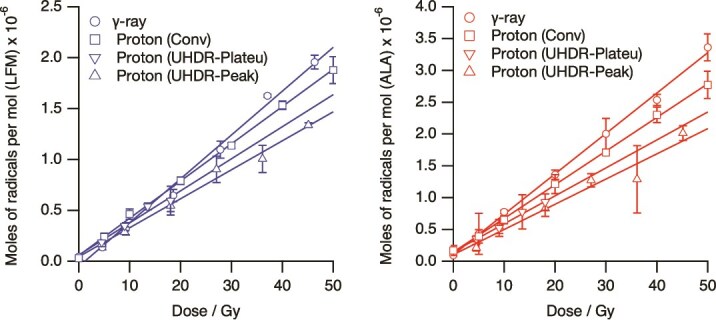
Concentrations of radicals to the physical dose of each radiation were plotted on the left for LFM and on the right for ALA, respectively. Vertical values are mol number of radicals in 1 mol of LFM or ALA.

Concentrations of radicals to the physical dose of each radiation are plotted in Fig. 2 left for LFM and right for ALA, respectively. Vertical values are mol number of radicals in 1 mol of LFM or ALA. The change in radical concentration with increasing dose for both LMF and ALA was linear for all radiation qualities, showing excellent linearity. The range of these error bars encompasses the portion reflecting the dose error, as well as errors in ESR measurements due to slight differences in the sample’s position within the ESR cavity. The slope of this graph, which is the number of radical moles produced per absorbed dose, the relative radical production efficiency when the slope for the γ-ray case is 1.00 as RE (the relative effectiveness), and goodness of fit (*R*^2^ values) are shown in Tables 2 and 3. For the same quality of radiation, the slope values were ~1.4 times higher for ALA than for LFM. This result indicates that the number of radicals produced per absorbed dose is greater with ALA than with LFM; however, as will be discussed later, this does not imply that ALA is superior to LFM as a chemical dosimeter. The RE values for both LFM and ALA decreased in the following order: γ-rays, CONV irradiation, ultra-high dose radiation of proton in the plateau region (UHDR-Plateau), and ultra-high dose radiation of proton in the peak region (UHDR-Peak). Henriksen *et al.* conducted heavy ion irradiation of alanine in 1966, reporting that the yield of free radicals decreased with increasing LET [15]. Later, Hansen and others also confirmed that the radical yield in alanine decreases as the LET increases [17, 18, 24]. For 16 and 6 MeV proton beams with LETs of 2.7 and 6.8 keV μm^−1^, the REs are reported to be 1.00 and 0.85, respectively. Fattibene *et al.* reported proton irradiation of alanine in the energy range of 1.6–6.1 MeV and a decrease in RE value from 0.623 to 0.607 as the LET increased from 6.630 to 18.032 keV μm^−1^ [21]. Onori *et al.* irradiated an ALA with a 62 MeV proton beam and compared the results using a Markus parallel plate ion chamber for dose evaluation. Although there is no recorded value for the LET at the irradiation position, the results of both methods were nearly identical, and it was reported that the RE value of the ALA was nearly unity [20]. In proton irradiation at LFM, it has been reported that the RE value in proton irradiation is 0.86 compared to X-ray irradiation [42]. Krushev *et al.* concluded that the relationship between the increase in LET and the decrease in the yield of free radicals in ALA was due to an increase in radical–radical recombination reactions caused by an increase in the local dose and the collapse of radical trapping sites [53]. Gu *et al.* have reported that ALA dosimetry for FLASH irradiation of X-ray (8 × 10^3^ Gy/s) and electron beam (8 × 10^8^ Gy/s) as an instantaneous dose rate and concluded that the ALA dosimeter showed no dose rate dependence both for X-ray and electron beam FLASH irradiation [34], including that UHDR with low LET rays does not lead to a decrease in RE values. If the RE values of LFM or ALA irradiated with protons in this study depend on LET only, then the RE values should be ranked in the order of UHDR-Plateau, CONV and UHDR-Peak. However, the RE values were higher for CONV with an LET of 2.0 keV μm^−1^ than for UHDR-Plateau with an LET of 1.0 keV μm^−1^. Although the LET in the UHDR-Plateau region was approximately half that of CONV (1.0 vs 2.0 keV/μm), the dose rate was >10 000 times higher. This unusual combination—a low LET with an extremely high dose rate—may promote localized radical density and enhance radical-radical recombination, ultimately reducing the number of detectable radicals. It has been confirmed both experimentally and theoretically that the generation of radical species decreases as the dose rate increases under low LET irradiation in the radiolysis of water [54–56]. Consequently, it is hypothesized that these factors contribute to the collapse of crystal structures and the recombination of generated radicals, resulting in the RE of UHDR-Plateau being less than that of CONV. Although the difference was minimal, the decrease in the RE value was somewhat less for LFM than for ALA. This inconsistency suggests that factors beyond physical LET may influence RE values under UHDR conditions. It could be related that the reaction paths to produce ALA radicals by radiation were complicated [26, 29, 32, 33], while those of LFM radical might be relatively simple. Another contributing factor may be limitations in the spatial resolution and accuracy of LET simulation, especially under steep gradients in narrow SOBPs. Moreover, the concept of ‘effective LET’, incorporating the dynamic interplay between energy deposition and radical kinetics under ultrafast irradiation conditions, may be more appropriate to explain the observed reduction in radical yield. Further investigations combining high-resolution dosimetry, improved LET mapping and real-time radical detection techniques will be essential to clarify the mechanisms behind this unexpected RE behavior.

**Table 2:** Number of generated radicals per absorbed dose of LFM for each beam and linearity

Radiation	Slope (nmol/Gy)	RE^a^	*R* ^2^
γ-Rays	43.1	1.000	0.9913
Proton CONV	36.6	0.849	0.9989
Proton (UHDR-Plateau)	31.5	0.731	0.9600
Proton (UHDR-Peak)	28.5	0.661	0.9876

^a^The relative effectiveness, RE, of high-LET radiation as the ratio between the radiation sensitivity for high-LET radiation and that for low-LET (^60^Co γ-ray) radiation.

**Table 3:** Number of generated radicals per absorbed dose of ALA for each beam and linearity

Radiation	Slope (nmol/Gy)	RE	*R* ^2^
γ-Rays	63.5	1.000	0.9976
Proton CONV	52.9	0.834	0.9994
Proton (UHDR-Plateau)	44.0	0.692	0.9951
Proton (UHDR-Peak)	39.6	0.624	0.9525

The *R*^2^ value exceeded 0.99 for LFM with both γ-ray and conventional proton beam irradiation and for ALA during UHDR irradiation in the plateau region, indicating good linearity. In the case of UHDR irradiation of the plateau and peak regions of LFM, and in the case of UHDR irradiation of the peak region of ALA, the value of *R*^2^ was <0.99. While the *R*^2^ value for the plateau region of FLASH irradiation is the lowest for LFM, it is close to 0.99 for the peak region. One reason for the smaller *R*^2^ value in the case of UHDR compared to CONV may be the difference in dose uniformity. For UHDR, the dose uniformity is estimated to be ~15 mm in the XY position and 10 mm in depth for each well of the 96-well plate. When placing the 96-well plate containing the samples on the irradiation stand, if the angle in the XY position is slightly off, the actual dose will differ for the three samples that should have been irradiated with the same dose, and there is a possibility that this will reduce the linearity of the peak intensity with respect to the physical dose.

**Fig. 3:**
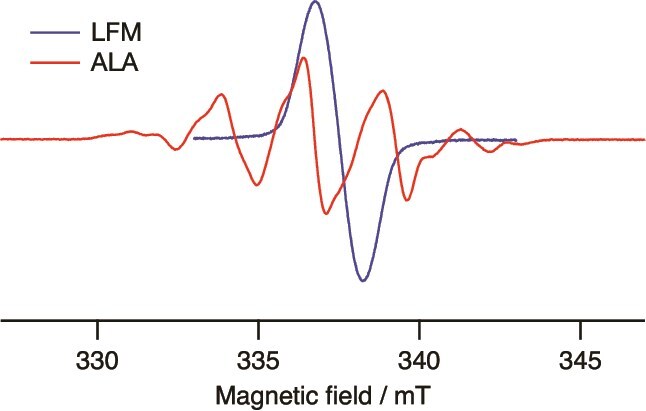
ESR spectra of -irradiated LFM (blue) and ALA (red) at room temperature with equimolar radicals.

As shown in Tables 2 and 3, the amount of residual free radicals is ~1.4 times higher for ALA than for LFM at the same dose and radiation quality. However, if the line width of the ESR signal remains unchanged with the dose, the peak height serves as an indicator of the sensitivity of these ESR chemical dosimeters. The quantity of radicals is proportional to the double integral of the ESR signal. Under our ESR measurement conditions, if the number of radicals produced in the LFM and ALA were equal, the height of the LFM signal would be 1.8 times greater than the height of the central peak of the ALA (Fig. 3). There were no significant differences in the line width or spectrum shape of the ESR spectra of the free radicals generated during LFM and ALA with γ-rays, CONV and UHDR. The line widths broaden as the radical density increases, but this was not observed in this analysis. It is believed that this occurs because the recombination reaction between radicals, which caused the decrease in RE values, progressed during proton irradiation, leading to a reduction in radical density in the crystal to a level comparable to that during γ-ray irradiation. Table 4 shows the ratio of the height of the central peak of LFM to that of ALA for each type of radiation. When the horizontal axis represents the dose and the vertical axis represents the peak height, the relative values of the REs observed from the slopes for each irradiation, with the slope of the γ-ray-irradiated LFM set to 1.00, are detailed in Table 4. For all types of radiation, the height of the central peak is ~20% higher for LFM than for ALA. The ESR signal of the irradiated LFM is a monotonous singlet line, and the magnetic field sweep width is only about half that of ALA. It indicates that LFM has the potential to be an ESR chemical dosimeter with higher sensitivity than ALA, not only for γ-rays but also for proton irradiation of CONV and UHDR.

**Table 4:** Sensitivity of LFM and ALA dosimeters to each type of radiation on the basis of relative peak height

Radiation	Relative height	
	LFM	ALA
γ-Rays	100.0	81.9
Proton CONV	84.9	68.2
Proton (UHDR-Plateau)	73.1	56.7
Proton (UHDR-Peak)	66.1	51.0

In this study, we irradiated LFM and ALA with a proton beam under UHDR conditions for the first time in the world and demonstrated the potential of UHDR conditions as a chemical dosimeter. However, we have not yet investigated the fading of ESR signals in both LFM and ALA after proton UHDR irradiation. Fading has been reported for both LFM [57, 58] and ALA [14, 18] in proton irradiation using CONV. Further investigation is necessary to use LFM and ALA as ESR chemical dosimeters more accurately.

## References

[1] Wilson JD, Hammond EM, Higgins GS, Petersson K. Ultra-high dose rate (FLASH) radiotherapy: silver bullet or fool's gold? (vol 9, 1563, 2020). Front Oncol 2020;10:210. 10.3389/fonc.2020.00210. 32161721 PMC7053480

[2] Wilson JD, Hammond EM, Higgins GS, Petersson K. Ultra-high dose rate (FLASH) radiotherapy: silver bullet or fool's gold? Front Oncol 2020;9:1563. 10.3389/fonc.2019.01563. 32010633 PMC6979639

[3] Berry RJ, Hall EJ, Forster DW et al. Survival of mammalian cells exposed to X rays at ultra-high dose-rates. Brit J Radiol 1969; 42:102–7. 10.1259/0007-1285-42-494-102. 4975207

[4] Berry RJ, Stedeford JB. Reproductive survival of mammalian-cells after irradiation at ultra-high dose-rates: further observations and their importance for radiotherapy. Brit J Radiol 1972;45:171–7. 10.1259/0007-1285-45-531-171. 5015264

[5] Weiss H, Epp ER, Heslin JM et al. Oxygen depletion in cells irradiated at ultra-high dose-rates and at conventional dose-rates. Int J Radiat Biol 1974;26:17–29. 10.1080/09553007414550901. 4607987

[6] Weiss H, Epp ER, Ling CC et al. Oxygen depletion in cells irradiated at ultra-high dose-rates and at conventional dose-rates. Radiat Res 1974;26:17–29. https://www.jstor.org/stable/3573881. 10.1080/09553007414550901 4607987

[7] Favaudon V, Caplier L, Monceau V et al. Ultrahigh dose-rate FLASH irradiation increases the differential response between normal and tumor tissue in mice. Sci Transl Med 2014;6:245ra93. 10.1126/scitranslmed.3008973. 25031268

[8] Vozenin MC, De Fornel P, Petersson K et al. The advantage of FLASH radiotherapy confirmed in mini-pig and cat-cancer patients. Clin Cancer Res 2019;25:35–42. 10.1158/1078-0432.CCR-17-3375. 29875213

[9] Common Terminology Criteria for Adverse Events (CTCAE) v5.0 . https://ctep.cancer.gov/protocolDevelopment/electronic_applications/ctc.htm#ctc_50 (April 11 2025, date last accessed).

[10] Bourhis J, Sozzi WJ, Jorge PG et al. Treatment of a first patient with FLASH-radiotherapy. Radiother Oncol 2019;139:18–22. 10.1016/j.radonc.2019.06.019. 31303340

[11] Miyagawa I, Gordy W. Electron spin resonance of an irradiated single crystal of alanine: second-order effects in free radical resonances. J Chem Phys 1960;32:255–63. 10.1063/1.1700912.

[12] Morton JR, Horsfield A. Electron spin resonance spectrum and structure of CH_3_ĊH(CO_2_H). J Chem Phys 1961;35:1142–3. 10.1063/1.1701206.

[13] Bradshaw WW, Spetzler HA, Cadena DG et al. Use of alanine as a solid dosimeter. Radiat Res 1962;17:11–21. 10.2307/3571206. 13872350

[14] Ebert PJ, Hardy KA, Cadena DG. Energy dependence of free radical production in alanine. Radiat Res 1965;26:178–97. 10.2307/3571776. 4284205

[15] Henriksen T . Production of free radicals in solid biological substances by heavy ions. Radiat Res 1966;27:676–93. 10.2307/3571850. 4286872

[16] Henriksen T . Effect of irradiation temperature on production of free radicals in solid biological compounds exposed to various ionizing radiations. Radiat Res 1966;27:694–709. 10.2307/3571851. 4286873

[17] Hansen JW, Olsen KJ. Theoretical and experimental radiation effectiveness of the free-radical dosimeter alanine to irradiation with heavy charged-particles. Radiat Res 1985;104:15–27. 10.2307/3576774.

[18] Hansen JW, Olsen KJ, Wille M. The alanine radiation detector for high and low LET dosimetry. Radiat Prot Dosim 1987;19:43–7.

[19] Gall K, Desrosiers M, Bensen D, Serago C. Alanine EPR dosimeter response in proton therapy beams. Appl Radiat Isot 1996;47:1197–9. 10.1016/S0969-8043(96)00201-1. 9022180

[20] Onori S, dErrico F, DeAngelis C et al. Alanine dosimetry of proton therapy beams. Med Phys 1997;24:447–53. 10.1118/1.597911. 9089596

[21] Fattibene P, De Angelis C, Onori S et al. Alanine response to proton beams in the 1.6-6.1 MeV energy range. Radiat Prot Dosim 2002;101:465–8. 10.1093/oxfordjournals.rpd.a006027. 12382792

[22] Palmans H . Effect of alanine energy response and phantom material on depth dose measurements in ocular proton beams. Technol Cancer Res Treat 2003;2:579–86. 10.1177/153303460300200610. 14640769

[23] Nattudurai R, Arous D, Edin NFJ, Malinen E. Alanine dosimeters for determining linear energy transfer in proton beams. Radiother Oncol 2021;161:S228–9. 10.1016/S0167-8140(21)07292-3.

[24] Hansen JW, Olsen KJ. Predicting decay in free-radical concentration in *L*-alpha-alanine following high-let radiation exposures. Appl Radiat Isot 1989;40:935–9. 10.1016/0883-2889(89)90019-1.

[25] Kuroda S, Miyagawa I. ENDOR study of an irradiated crystal of *L*-alanine: environment of the stable CH_3_ĊHCO_2_^−^ radical. J Chem Phys 1982;76:3933–44. 10.1063/1.443510.

[26] Sagstuen E, Sanderud A, Hole EO. The solid-state radiation chemistry of simple amino acids, revisited. Radiat Res 2004;162:112–9. 10.1667/RR3215. 15387137

[27] Minegishi A, Shinozaki Y, Meshitsuka G. Radiolysis of solid *L*-alpha-alanine. Bull Chem Soc Jpn 1967;40:1271–2. 10.1246/bcsj.40.1271. 4294170

[28] Minegishi A, Shinozaki Y, Meshitsuka G. Electron spin resonance spectra of an irradiated single crystal of *L*-alpha-alanine at 77 degrees K. Bull Chem Soc Jpn 1967;40:1549–50. 10.1246/bcsj.40.1549. 4294171

[29] Sagstuen E, Hole EO, Haugedal SR, Nelson WH. Alanine radicals: structure determination by EPR and ENDOR of single crystals X-irradiated at 295 K. J Phys Chem A 1997;101:9763–72. 10.1021/jp972158k.

[30] Heydari MZ, Malinen E, Hole EO, Sagstuen E. Alanine radicals. 2. The composite polycrystalline alanine EPR spectrum studied by ENDOR, thermal annealing, and spectrum simulations. J Phys Chem A 2002;106:8971–7. 10.1021/jp026023c.

[31] Malinen E, Heydari MZ, Sagstuen E, Hole EO. Alanine radicals, part 3: properties of the components contributing to the EPR spectrum of X-irradiated alanine dosimeters. Radiat Res 2003;159:23–32. 10.1667/0033-7587(2003)159[0023:ARPPOT]2.0.CO;2. 12492365

[32] Pauwels E, De Cooman H, Waroquier M et al. Solved? The reductive radiation chemistry of alanine. Phys Chem Chem Phys 2014;16:2475–82. 10.1039/C3CP54441A. 24356118

[33] Jåstad EO, Torheim T, Villeneuve KM et al. In quest of the alanine R3 radical: multivariate EPR spectral analyses of X-irradiated alanine in the solid state. J Phys Chem A 2017;121:7139–47. 10.1021/acs.jpca.7b06447. 28829916

[34] Gu RQ, Wang JL, Wang P et al. Alanine/electron spin resonance dosimetry for FLASH radiotherapy. Radiat Phys Chem 2024;225:112113. 10.1016/j.radphyschem.2024.112113.

[35] Lund A, Olsson S, Bonora M et al. New materials for ESR dosimetry. Spectrochim Acta, Pt A: Mol Biomol Spectrosc 2002;58:1301–11. 10.1016/S1386-1425(01)00719-3. 11993477

[36] Waldeland E, Hole EO, Sagstuen E, Malinen E. The energy dependence of lithium formate and alanine EPR dosimeters for medium energy X rays. Med Phys 2010;37:3569–75. 10.1118/1.3432567. 20831064

[37] Adolfsson E, Wesolowska P, Izewska J et al. End-to-end audit: comparison of TLD and lithium formate EPR dosimetry. Radiat Prot Dosim 2019;186:119–22. 10.1093/rpd/ncy289. 30929009

[38] Waldeland E, Hole E, Sagstuen E et al. Dosimetry of ion beams using lithium formate EPR dosimeters. Radiother Oncol 2005;76:S122. 10.1016/S0167-8140(05)81234-4.

[39] Costa T, Adolfsson E, Fager M, Lund E. Characterization of a lithium formate EPR-dosimetry system for proton radiation therapy. Radiat Prot Dosim 2019;186:83–7. 10.1093/rpd/ncy293. 30624734

[40] Lund E, Gustafsson H, Danilczuk M et al. Compounds of ^6^Li and natural Li for EPR dosimetry in photon/neutron mixed radiation fields. Spectrochim Acta, Pt A Mol Biomol Spectrosc 2004;60:1319–26. 10.1016/j.saa.2003.10.029. 15134730

[41] Alejandro G, Longhinoh J, Alvarez R et al. A dual natural lithium formate/L-alanine EPR dosimeter for a mixed radiation field in a boron neutron capture therapy irradiation facility. J Phys D Appl Phys 2020;53:165001. 10.1088/1361-6463/ab6e45.

[42] Belahmar A, Mikou M, Hoehr C, el Ghalmi M. Cumulative dose experiments on lithium formate monohydrate as an EPR-dosimeter for use in different radiation therapy scenarios. Nucl Inst Methods Phys Res B 2022;532:1–6. 10.1016/j.nimb.2022.10.001.

[43] D'Oca MC, Collura G, Gagliardo C et al. Improvement of neutron sensitivity for lithium formate EPR dosemeters: a Monte Carlo analysis. Radiat Prot Dosim 2023;199:1591–9. 10.1093/rpd/ncac268. 37721086

[44] Vestad TA, Gustafsson H, Lund A et al. Radiation-induced radicals in lithium formate monohydrate (LiHCO_2_•H_2_O). EPR and ENDOR studies of X-irradiated crystal and polycrystalline samples. Phys Chem Chem Phys 2004;6:3017–22. 10.1039/B402846E.

[45] Komaguchi K, Matsubara Y, Shiotani M et al. An ESR and ENDOR study of irradiated Li-formate. Spectrochim Acta, Pt A: Mol Biomol Spectrosc 2007;66:754–60. 10.1016/j.saa.2006.04.023. 16875868

[46] Krivokapic A, Aalbergsjo SG, De Cooman H et al. Lithium formate for EPR dosimetry: radiation-induced radical trapping at low temperatures. Radiat Res 2014;181:503–11. 10.1667/RR13582.1. 24720752

[47] Krivokapic A, Sanderud A, Aalbergsjo SG et al. Lithium formate for EPR dosimetry (2): secondary radicals in X-irradiated crystals. Radiat Res 2015;183:675–83. 10.1667/RR14046.1. 26010706

[48] Iwata H, Ogino H, Hashimoto S et al. Spot scanning and passive scattering proton therapy: relative biological effectiveness and oxygen enhancement ratio in cultured cells. Int J Radiat Oncol Biol Phys 2016;95:95–102. 10.1016/j.ijrobp.2016.01.017. 27084632

[49] Toshito T, Omachi C, Kibe Y et al. A proton therapy system in Nagoya proton therapy Center. Australas Phys Eng Sci Med 2016;39:645–54. 10.1007/s13246-016-0456-8. 27271800

[50] Iwata H, Akita K, Yamaba Y et al. Concurrent chemo-proton therapy using adaptive planning for unresectable stage 3 non-small cell lung cancer: a phase 2 study. Int J Radiat Oncol Biol Phys 2021;109:1359–67. 10.1016/j.ijrobp.2020.11.035. 33227444

[51] Iwata H, Toshito T, Omachi C et al. Proton FLASH irradiation using a synchrotron accelerator: differences by irradiation positions. Int J Radiat Oncol Biol Phys 2025;121:1293–302. 10.1016/j.ijrobp.2024.11.066. 39549758

[52] Nomura K, Iwata H, Toshito T et al. Biological effects of passive scattering and spot scanning proton beams at the distal end of the spread-out Bragg peak in single cells and multicell spheroids. Int J Radiat Biol 2021;97:695–703. 10.1080/09553002.2021.1889704. 33617430

[53] Krushev VV, Koizumi H, Ichikawa T et al. Relation between track structure and LET effect on free-radical formation for ion beam-irradiated alanine dosimeter. Radiat Phys Chem 1994;44:521–6. 10.1016/0969-806X(94)90051-5.

[54] Trumbore CN, Youngblade W, Short DR. Computer modeling of data from pulse radiolysis studies of aqueous solutions containing scavengers of spur intermediates. J Phys Chem 1984;88:5057–61. 10.1021/j150665a052.

[55] Shiraishi H, Katsumura Y, Hiroishi D et al. Pulse-radiolysis study on the yield of hydrated electron at elevated temperatures. J Phys Chem 1988;92:3011–7. 10.1021/j100321a061.

[56] Alanazi A, Meesungnoen J, Jay-Gerin JP. A computer modeling study of water radiolysis at highdose rates. Relevance to FLASH radiotherapy. Radiat Res 2021;195:149–62. 10.1667/RADE-20-00168.1. 33300999

[57] Waldeland E, Hole EO, Stenerlöw B et al. Radical formation in lithium formate EPR dosimeters after irradiation with protons and nitrogen ions. Radiat Res 2010;174:251–7. 10.1667/RR2035.1. 20681791

[58] Adolfsson E, Karlsson M, Carlsson GÅ et al. Investigation of signal fading in lithium formate EPR dosimeters using a new sensitive method. Phys Med Biol 2012;57:2209–17. 10.1088/0031-9155/57/8/2209. 22456424

